# Perspective: Hollow Core Optical Fibres for Ultraviolet and Visible Wavelengths

**DOI:** 10.1002/advs.202517476

**Published:** 2026-01-20

**Authors:** Robbie Mears, Kerrianne Harrington, Jonathan C. Knight, William J. Wadsworth, James M. Stone, Tim A. Birks

**Affiliations:** ^1^ Centre for Photonics, Department of Physics University of Bath Bath UK

**Keywords:** anti‐resonant, hollow core, ultraviolet, visible

## Abstract

Anti‐resonant hollow core fibres have significant advantages derived from the guidance of light in air, and have been rapidly developed and employed in the near and mid‐infrared. Despite comparable or greater benefits in the visible and ultraviolet, their development and uptake has been slow due to inherent challenges in their fabrication. In this perspective, we provide a brief look at these fabrication challenges, possible solutions and potential applications for hollow core fibres in the short‐wavelength limit.

## Graphical abstract

Hollow core optical fibres bypass material constraints that limit optical fibres at ultraviolet and visible wavelengths. However, their challenging fabrication has limited their development, and significant gains in performance remain possible. In this perspective we outline approaches to enable the next generation of fibres for shorter wavelengths, with applications in photolithography, environmental sensing, and vacuum‐UV technologies.

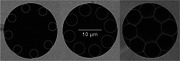

## 1 Introduction

Ultraviolet and visible (UV/Vis) wavelengths of light are widely used in a range of fields, including photolithography [1], microscopy [2] and environmental sensing [3, 4]. Their utility stems from the higher energy and shorter wavelengths than the near‐infrared, enabling stronger light–matter interactions over smaller scales. Many materials exhibit strong absorption features in the ultraviolet and visible spectral regions, and as a result, spectroscopic techniques such as ultraviolet and visible (UV/Vis) absorption spectroscopy [5, 6] are widely employed across industry and academia to probe electronic transitions and structural characteristics.

For many of these applications, these same absorptions can pose significant technological challenges, limiting the available spectral range and tolerable powers. This is especially true for optical fibres, which exhibit rapidly increasing losses from Rayleigh scattering and electronic absorptions, yielding total losses of ∼1 dB/m by 200 nm wavelength [7]. Even worse, on exposure to intense visible or ultraviolet light a process called solarization can occur in fibres, where the breaking of inter‐atomic bonds by the intense light creates absorbing colour centers [8], further increasing absorption losses. While conventional solid core optical fibres are still widely used at ultraviolet wavelengths they remain limited in their spectral range, power handling and loss.

These fundamental material limitations are handily avoided in hollow core fibres, where light propagates through a gas or vacuum. At wavelengths from the ultraviolet to the mid‐infrared, silica‐based hollow core fibres now have lower attenuation than their solid core counterparts [9, 10, 11, 12]. Even in the near‐infrared, where solid silica fibres are at their optimal, hollow core fibres now show the lowest loss by a significant margin [13]. Beyond loss, other benefits such as the ultra‐low non‐linearity [14], low latency [15] and high damage thresholds [16] have enabled a range of applications that were not previously possible using conventional solid core fibres.

These advantages of hollow core guidance grow as the material performance of conventional fibres decreases [17], particularly toward shorter wavelengths in the ultraviolet and visible (UV/Vis). In hollow core fibres, these effects are greatly suppressed and fibre losses of 10–1000 times lower than any solid core fibre can be achieved [9, 10], a larger advantage than possible in the near‐infrared [17].

A further advantage enjoyed by silica hollow core fibre at these shorter wavelengths is the lack of suitable conventional fibre materials, in contrast to mid‐infrared wavelengths. At those longer wavelengths, there are many alternative glasses [18] that show greatly improved mid‐infrared transparency and extend the operational spectral range of solid core fibres. This reduces the utility of silica based hollow core fibres, which become absorption‐loss limited around ∼3.5 μm [11] wavelength. However, at visible and ultraviolet wavelengths the materials with improved transparency, such as ${\mathrm{MgF}}_{2}$, ${\mathrm{CaF}}_{2}$ or Sapphire are crystalline and cannot be drawn into optical fibres with conventional fibre fabrication techniques. Even if an improved material were found, it would be almost impossible to reach the level of technological maturity that fused silica exhibits. With decades of development for telecommunications applications, large quantities of ultra‐pure silica glass are readily available commercially, at low cost, in a range of sizes suitable for hollow core fibre fabrication.

Despite the large potential benefits, lack of alternatives and long established need, the development of UV/Vis guiding hollow optical fibres has lagged behind their near‐infrared counterparts. At telecommunications wavelengths, hollow core fibres are now reported with ultra‐low losses [13, 19], strongly single mode guidance [20] and bend resistance competitive with conventional solid core fibres [21]. In contrast, reports of UV/Vis guiding fibres are sporadic and have yet to approach fundamental limits. Compared to the near‐infrared, these fibres display narrow bandwidths, higher losses and much stronger bend sensitivity. The highly successful nested structures which have enabled the lowest loss optical fibres to date [13, 19], have yet to be replicated at UV/Vis wavelengths. A parametric optimization [17] of such a nested fibre suggests that fibres with total losses of 10–1 dB/km should be possible from 200–700 nm, far below most current fibres in that wavelength range. Finally, the vacuum‐ultraviolet range of ∼100–200 nm remains practically unexplored beyond some promising initial transmission measurements [22] and a few non‐linear experiments over centimeter length scales [23, 24]. At these wavelengths, no practical optical fibres currently exist and so any fibre with reasonable propagation and bend losses would be transformational.

In this perspective, we argue that this asymmetry of development is due to the fabrication challenges associated with anti‐resonant hollow core fibres, which affect UV/Vis guiding fibres to a greater degree. We identify a series of fabrication advances and modifications that could enable a new generation of optical fibres for visible and ultraviolet that are currently impractical or impossible to fabricate. Finally, we briefly outline key applications that would benefit greatly from the new fibres.

## 2 Current Challenges in UV/Vis Guiding Hollow Core Fibres

Anti‐resonant hollow core fibres guide light through a series of grazing incidence reflections from a number of thin glass capillaries that surround a hollow central core region. These glass capillaries are carefully engineered to ensure the destructive (anti‐resonant) interference of light transmitted through their thin walls, such that their reflectivity is greatly enhanced, reducing leakage losses. While a full understanding of the guidance within these fibres requires additional effects such as azimuthal confinement [25] and inhibited coupling [26], anti‐resonance can be considered a necessary but not sufficient condition for low loss guidance. The destructive and constructive (resonant) interference conditions are well described by the ARROW model [27], which predicts the wavelength $\lambda_{m}$ of resonant bands

(1) $$\lambda_{m}=\frac{2t}{m}{\left(n^{2}-1\right)}^{\frac{1}{2}}$$

where resonance order *m* is an integer on resonance, *t* is the wall thickness and *n* is the refractive index of silica. This results in a series of high‐loss resonances for integer values of *m* (m=1,2,3,…) with wide low‐loss ranges in between (around anti‐resonances m=1/2,3/2,5/2,…). From this simple relationship we see that guidance at shorter wavelengths requires thinner capillary walls *t* and/or higher anti‐resonant orders *m*. However, higher order bands have reduced bandwidth due to more closely separated resonances and also have increased overlap of light and glass, which increases absorption losses [17]. If possible, it is therefore usually preferred to guide in lower order bands, such as the m=3/2 or even m=1/2 bands.

Just as with the capillary wall thickness, the central core region should also be a certain size for optimal fibre performance. While larger core diameters reduce leakage, scattering and absorption losses, the opposite trend applies for microbending and macrobending loss mechanisms, which worsen with larger core diameters. The relative importance of different loss mechanisms, particularly absorption and scattering losses, also depends on the wavelength of interest. As such, the optimal core diameter will depend on the intended application and operating wavelengths. For the lowest total loss, a parametric optimization can be performed, but generally good performance is achieved in designs with core diameters of ∼20–30 times the guided wavelength [9, 28, 29, 30].

**FIGURE 1:**
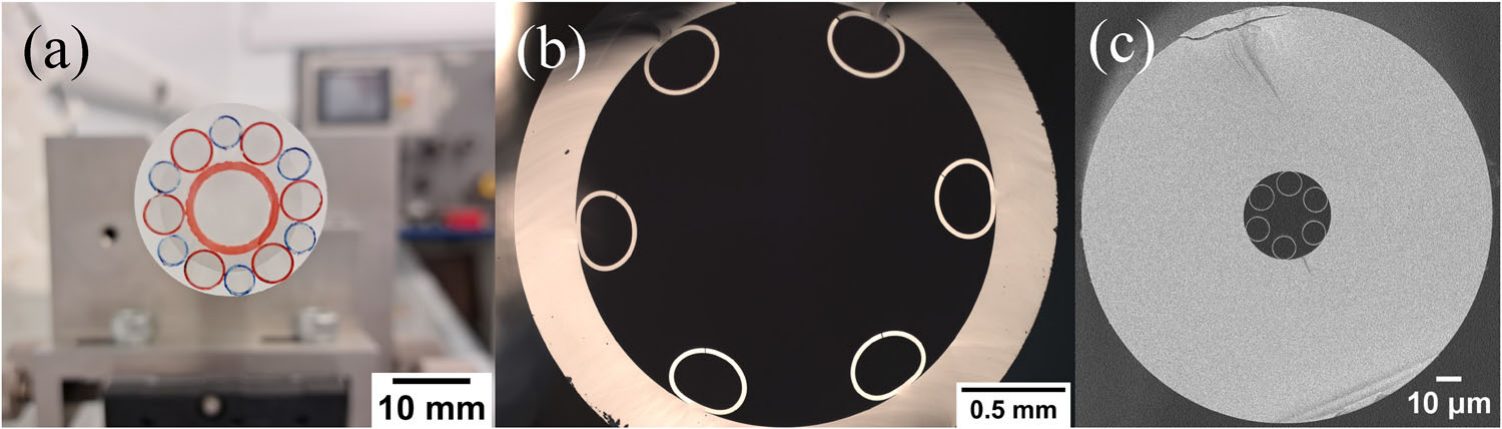
Stack and draw fabrication process. (a) A photograph showing the end face of a 6 capillary stack, where blue rings are the fibre capillaries and red highlighted rings are supports that only extend partially through the stack. (The colours are inked onto the transparent glass tubes for illustrative purposes.) (b) Optical micrograph of a cane drawn from the stack in (a). (c) A fibre drawn from one of the set of canes in (b).

The fabrication of practically all anti‐resonant hollow core fibres takes place using the stack and draw technique [31] where a macroscopic “preform” version of the fibre is created by stacking millimeter‐scale elements, before being heated and drawn under tension, usually in several steps, down to the microscopic fibre scale. In Figure 1 examples of each stage of this process are shown schematically, from the initial stack to the final fibre.

**FIGURE 2:**
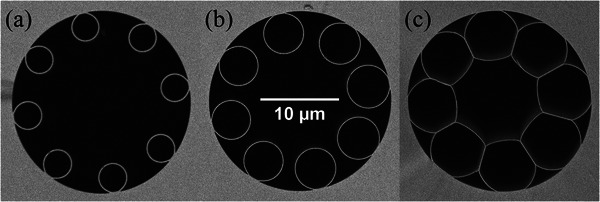
SEMs of 8 capillary fibres with (a) underinflated capillaries (b) well inflated capillaries and (c) contacting (overinflated) capillaries.

The fundamental challenge in this fabrication process is controlling the size and thickness of inherently unstable glass capillaries during fibre drawing, when the heated glass is fluid. These thin capillaries experience strong surface tension forces that tend to cause their contraction, which must be carefully counterbalanced by an applied internal pressure. If too little pressure is applied then the capillaries contract and the final fibre experiences high loss through the large inter‐capillary gaps. An equally undesirable outcome occurs with excessive pressure, as capillaries will contact and experience high loss through the thicker contact points. These outcomes are shown in Figure 2 for a UV/Vis guiding fibre.

Due to this delicate balancing act, there is a finite pressure range over which the desired structure can be successfully drawn, as in Figure 2b. As this pressure range become smaller, a particular fibre becomes increasingly difficult and eventually impossible to fabricate [32]. For hollow core fibres at shorter wavelengths, the smaller structures and thinner capillaries are naturally more unstable and greatly narrow this pressure range [33]. This is a simplification of the full dynamics at play, which are described comprehensively in [32].

The greater fabrication challenge at shorter wavelength has largely prevented a strict scaling of structures from the near‐infrared to UV/Vis wavelengths. In the few works that have attempted such a scaling [34, 35], the reported fibres had asymmetric structures that were only drawn in short lengths, showing their fabrication was a clear limitation in the final fibre performance. At the same time, kilometer lengths of more complex nested structures with losses at the dB/km [36] level were beginning to be reported in the near‐infrared, highlighting how this fabrication challenge was specific to these shorter wavelength scales.

**FIGURE 3:**
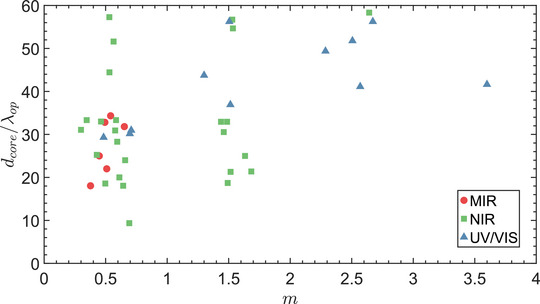
A plot of the ratio of core diameter to operating wavelength vs. resonance order *m* for various fibres reported in the literature [2, 9, 10, 11, 13, 16, 20, 29, 30, 34, 36, 37, 38, 39, 40, 41, 42, 43, 44, 45, 46, 47, 48, 49, 50, 51, 52, 53, 54, 55, 56, 57, 58, 59, 60, 61, 62]. The reported fibres cluster around half‐odd‐integer, corresponding to anti‐resonance. The parameters for each fibre were either given in the cited text or determined using provided images and data. The spectral regions were labeled as follows: UV/Vis (< 700 nm), near‐IR (700–3000 nm), and mid‐IR (>3000 nm).

The consequences of the fabrication challenges that lead to this asymmetry can be seen clearly in Figure 3, where the ratio of core diameter $d_{core}$ to operating wavelength $\lambda_{op}$ for reported hollow core fibres is plotted against the resonance band m. The 43 fibres were chosen to be a representative sample of the literature, with a range of applications at wavelengths from the ultraviolet to the mid‐infrared.

At near‐infrared wavelengths, the core diameters of reported fibres can vary greatly from 10–60 times the guided wavelength, satisfying a wide range of different applications. Most low loss fibres have core diameter to wavelength ratios of 20–30 to balance various confinement, scattering and bending losses. The flexibility and optimization possible for near‐ and mid‐infrared guiding fibres is almost entirely lost in UV/Vis guiding fibres, which have much larger core diameter ratios of 40–60. This limits the range of possible applications and yields generally higher bend sensitivity.

The anti‐resonant band order is similarly constrained, with a majority of UV/Vis fibres guiding in higher order m=3/2 or m=5/2 bands. These fibres are easier to fabricate because they have thicker capillary walls but they provide limited bandwidths compared to the broad fundamental m=1/2 band that the majority of near‐infrared and mid‐infrared fibres guide within. At mid‐infrared wavelengths, the much easier fabrication allows practically all fibres to be fabricated with the optimal core diameter and fundamental band guidance, as seen by the tight cluster of reported fibre parameters.

In summary, these fabrication challenges mean that UV/Vis fibres have not yet reached their fundamental loss and bandwidth limits. At near‐infrared wavelengths, the use of nested elements has greatly reduced confinement losses ‐ leaving surface scattering and microbending losses as the main loss mechanisms for state of the art fibres [13, 19]. As these losses have opposite scaling with core diameter [17], a minimum total loss at a given wavelength is achieved for a specific core diameter [63] which carefully balances the two losses. This approach has yet to be employed at UV/Vis wavelengths, where fabrication challenges have thus far prevented nested elements and appropriately sized core diameters. The large core diameters of reported fibres likely yields an unequal proportion of losses, dominated by microbending, while surface scattering lies at a much lower level.

To highlight this, we have estimated the contribution of surface scattering to the losses of reported fibres. First, we take recent results at 1550 nm [13, 19], where surface scattering losses of around $\alpha_{SSL}$ = 0.05 dB/km are reported to be comparable to other sources of loss [19], to represent an optimum. Then we scale this value to other reported fibres using [64]

(2) $$\alpha_{SSL}=\eta F{\left(\lambda_{ref}/\lambda_{0}\right)}^{p}$$

where η is a constant, F the electric field amplitude intensity at the glass‐air boundary, $\lambda_{0}$ the free space wavelength and $\lambda_{ref}$ a reference wavelength. The constant *p* depends on the analysis used to reach this expression, with values of 2 [65], 2.5 [17] and 3 [10] all used in the literature. As recent work [64] has shown that *F* is well described by the simple relation $F\propto \frac{\lambda_{0}^{2}}{r_{c}^{3}}$, where $r_{c}$ is the radius of the core, Equation (2) can be simplified to

(3) $$\alpha_{SSL}\propto \lambda_{0}^{p-2}/r_{c}^{3}$$

**FIGURE 4:**
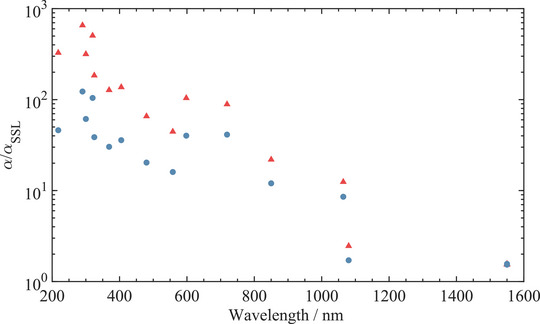
A plot of measured fibre losses α [9, 10, 13, 19, 34, 37, 40, 43, 45, 52, 66] divided by their estimated surface scattering loss $\alpha_{SSL}$ against wavelength. Red triangles correspond to *p* = 3 and blue circles *p* = 2.

The measured loss α of each reported fibre as a fraction of the estimated surface scattering loss $\alpha_{SSL}$ is plotted against wavelength in Figure 4. As there is still disagreement in the literature over the exact scaling of the surface scattering, we have included estimates for *p* values of 2 and 3 to represent the possible extremes.

The ratio of losses in this plot is a measure of how much each fibre's overall loss exceeds the contribution due to surface scattering. At near‐infrared wavelengths, where most other loss mechanism have been effectively controlled, the ratio approaches unity, and surface scattering is a large and unavoidable proportion of the measured loss. However, toward visible and ultraviolet wavelengths, the ratio exceeds 10 or even 100 as the estimated surface scattering loss becomes an increasingly insignificant fraction of the overall measured loss. Furthermore, for this comparison, we have assumed an unoptimized thermodynamically‐determined surface roughness across all fibres. If reported techniques to reduce this roughness [10] can indeed be applied, surface scattering could become a smaller proportion of the total, making its mitigation even less pressing until the other contributions are controlled.

Therefore, to improve the losses of UV/Vis fibres, we must first focus on reducing confinement and microbending losses rather than surface scattering loss. For confinement loss, the use of nested elements drastically reduces leakage losses and enables low loss operation at much smaller core diameters [21] than possible in fibres without nested elements. Smaller core diameters are key to reducing microbending losses, which have very strong dependences on the core diameter to wavelength ratio [17]. Beyond the internal structure, larger fibre outer diameters and carefully chosen coatings, which mechanically stiffen the fibre, are also highly effective in mitigating microbending losses.

These strategies have been successfully employed for infrared fibres, and now need to be similarly employed for UV/Vis fibres. This entails the drawing of fibres with significantly thinner capillaries and smaller core diameters than have been previously reported. For fundamental band guidance (m=1/2) centered at wavelengths from 200–700 nm, this would require capillary wall thicknesses of ∼ 40–165 nm. The ideal core diameter is naturally more complicated as discussed previously, but values from 4–20 μm should satisfy the majority of applications in that same wavelength range. With state of the art fibres [9, 10, 30] all reporting wall thicknesses above 150 nm and core diameters exceeding 10 μm, reaching these ideal values requires a marked advance in fabrication capabilities. We now outline how this can be achieved.

## 3 Approaches and Solutions

The fabrication challenge associated with a specific fibre depends on its parameters, such as wall thickness, and drawing conditions such as furnace temperature. If the fibre parameters are fixed, then the only way to control the structure and ensure a reasonably sized pressure window is to carefully control the drawing conditions. An exception to this rule is a recently reported nested fibre [19], which used interstitial tubes to relax the requirement for small inter‐capillary gaps. As this greatly mitigates mid‐draw contact, we expect such a design to ease the fabrication challenges at UV/Vis wavelengths. However, in this perspective, we will focus on approaches that are generally applicable to all fibre designs. As explained in detail in [30], the main levers of control are the draw tension and drawdown ratio, which can both be manipulated to greatly ease the fabrication of challenging hollow core fibres.

A discussion around such parameters is greatly hindered by the general lack of fabrication parameters in the literature. For reported low loss solid and hollow core fibres [10, 13, 57, 67] their fabrication parameters are rarely given, or only partly so. This limits their reproducibility and impedes overall progress in the field. Without access to published values, researchers are forced to depend on institutional knowledge or trial‐and‐error approaches. As a result, even well‐resourced facilities may spend significant time rediscovering fabrication insights that others have already mastered, simply because those insights remain undocumented.

In that context, the following section uses the few published parameters in the literature and our current fabrication knowledge at the University of Bath.

### 3.1 Draw Tension

The draw tension is the force required to form a fibre from a softened preform during the drawing process. Its value is often given in the measured unit of grams but to enable the comparison of different fibres with coincidentally dissimilar outer diameters, we will instead refer to the draw stress with its units of MPa or GPa. The measured draw tension/stress depends on the glass viscosity and therefore the temperature. For a fibre drawn at a lower temperature, the glass is more viscous and therefore the capillaries respond more slowly to surface tension and pressure forces ‐ providing a wider pressure window over which a desired structure can be drawn. The practical limit to drawing at ever higher stresses is that of fibre breaks, where fracture of the drawn fibre occurs. These fibre breaks limit the maximum continuous length that can be practically drawn, and also waste material as the drawing process must be manually restarted after each fibre break. The chosen draw stress is thus a balance between controlling the capillaries and minimising the impact of these fibre breaks. For UV/Vis guiding fibres where higher stresses are needed to control the thin capillaries [32, 33], the draw stress must be maximized while maintaining an acceptable frequency of fibre breaks.

Fibre breaks are initiated by the presence of microscopic scratches and other surface flaws or defect sites, which greatly reduce the inherent strength of silica. To reduce the frequency of these breaks we therefore need to minimize the concentration of defects throughout the fabrication process. For solid core fibres, surface processing and high standards of cleanliness at all stages of fabrication mitigate surface defects and avoid potential contaminants. The success of this approach is evidenced by the routine proof‐testing of solid fibres up to GPa stresses over kilometer lengths [68, 69]. In contrast, the strength of fabricated hollow core fibres has yet to be explored in the literature, leaving questions as to the degree of cleanliness and ultimate strength of hollow core fibres.

A likely source of contamination is the current stack and draw method used for hollow core fibre fabrication. With a greater number of fabrication steps and increased surface area, the inclusion of contaminants from handling becomes more likely. Furthermore, in the final fibre draw stage, the use of glue or other sealants to differentially pressurize parts of the fibre can easily contaminate the entire preform if those sealants decompose or degrade at high temperatures. The usual processing steps of etching and flame polishing used for solid core fibres should improve these issues by removing contaminants and healing surface detects [70]. However, these methods currently only apply to external surfaces and in hollow core fibres, the internal surface area can be ∼30 % [9] to ∼60 % [13] of the total fibre surface area. As fibre breaks are a stochastic process, their investigation requires careful statistical analysis and none have yet been reported on the drawing strength of hollow core fibres. Nevertheless, generally improved cleanliness and surface treatments should permit higher drawing stresses without increasing the frequency of fibre breaks.

Another possible route to reduce the frequency of fibre breaks is to modify the distribution of the drawing stress. When drawing a fibre with a uniform viscosity profile, the entire structure experiences the same drawing stress and a fibre break may occur at any point in the structure. However, only the unstable capillaries require a high drawing stress, and the surrounding glass can be drawn at much lower stresses to reduce the overall probability of fibre breaks. A difference in viscosity and thus stress can be easily achieved through glass doping, for example, with fluorine, which reduces the softening point compared to pure silica. In the literature, there are only a few examples of this in action [28, 71] and its effectiveness has yet to be fully explored, but it may significantly reduce the frequency of fibre breaks for a given capillary drawing stress. A particularly interesting test case will be the fabrication of fibres where only the capillaries are non‐doped, as then only a very limited region will be under high drawing stress.

Along this same track, we propose that the stress distribution can similarly be manipulated through changes to process parameters only. We recently reported a transverse temperature gradient in the fabrication of a hollow core fibre [72]. In that work, the center of the preform was colder than its outer edges, which affected the inflation of the capillaries in the multi‐core structure. This uneven heating of the preform may be due to the speed at which the preform is drawn, and faster feed and draw rates should increase this gradient. While anecdotal, we have found that high throughput speeds do indeed allow higher tensions with a reduced frequency of breaks [9]. An example of this is the fibre used in [73], where the fibre was drawn using a feed rate of 70 mm per minute and a draw rate of ∼138 m per minute. A continuous length of 185 m of uniform fibre was drawn under these conditions despite a ∼95 nm wall thickness. In attempts to draw the same fibre at lower speeds, we experienced frequent fibre breaks and could not achieve the desired structure.

### 3.2 Drawdown Ratio

In a drawing process, the drawdown ratio is the ratio of the initial and final diameters involved, and its value determines the maximum yield as well as the fabrication difficulty. This scaling of difficulty with drawdown ratio occurs because the previously discussed balancing of capillary forces is naturally harder when the initial and final structures are more dissimilar in size.

For fibres guiding shorter wavelengths, this challenge is increased, as for a given starting preform, a smaller final structure naturally requires a larger drawdown ratio. In theory this provides a greater yield, but as described by [30], the increased instability from a larger drawdown ratio makes this hard to realize. To draw the desired structure, a high draw tension is required which then increases the probability of fibre breaks, negating the potentially increased yield. As this yield is unrealizable and only serves to enhance the fabrication difficulty a natural step to improve the fabrication of UV/Vis guiding fibres is to reduce the drawdown ratio.

For many hollow core fibres, the actual drawdown ratio is uncoupled from the final size by what is commonly referred to as a cane stage and shown in Figure 1b. Instead of directly drawing from the starting stack to the final fibre, an intermediate stage is introduced where the stack is drawn from a diameter of few cm (20–30 mm) to a cane diameter of a few mm (2–6 mm). The cane is then placed in a jacketing tube to increase its final size and then drawn to fibre. This reduction is useful for stabilising the final fibre draw and enables rapid iteration, as many canes can be drawn from a single stack with a high degree of uniformity and provide multiple attempts at fabrication without multiple stacks. The practical limit to ever smaller canes is the need to differentially pressurize the core and capillary regions. This is done through the use of various sealants, which are applied to the end of the cane by hand prior to drawing. With smaller canes, this becomes more difficult and eventually impractical.

A solution to both problems of drawdown and preform preparation is the inclusion of a second cane stage, described in detail in [30]. Briefly, this method consists of placing the first cane in a jacket as standard, before drawing to a smaller cane that is ultimately drawn to fibre. The key innovation in this method is retaining the transition region between the two cane stages, such that preform preparation can be done in the first and much larger cane. This provides the benefits of a smaller drawdown ratio without the need to apply sealants to small and delicate structures.

In their initial demonstration of this technique [30] the authors reported fibres with wall thicknesses of ∼ 167–254 nm, excellent uniformity and lengths up to 350 m. While impressive, the full potential of this method has yet to be realized. The ability to draw with an almost arbitrary drawdown ratio massively expands the available parameter space of both wall thickness and core diameter. In our recent works, we've been able to achieve wall thicknesses of ∼ 80–100 nm [72, 73] and core diameters ∼12 μm [9] using a single cane stage. With a two cane stage process, these fibres could be fabricated more easily and surpassed to achieve extreme wall thicknesses of 40–50 nm along with core diameters below 10 μm.

Finally, it should be noted that the smallest drawdown ratios are achieved in post‐processing techniques such as tapering [74, 75, 76] or etching [77, 78]. These techniques take readily available near‐infrared guiding fibres and scale to shorter wavelengths. In the case of etching, there is no further drawdown at all. In the case of tapering, drawdown proceeds incrementally in many small stages [79], minimizing the previously discussed fabrication limitations. As a result, the fibres with the thinnest wall thicknesses (∼60 nm [74]) and smallest core diameters (∼6 μm [76]) have been post‐processed. The disadvantage of these approaches are the inherently short lengths < 1 m that limits their use to short‐length applications.

## 4 Applications

To see the potential impact that a new generation of hollow core fibres could have at shorter wavelengths, we look at their applications in light delivery and non‐linear generation. While many more applications are possible, these have long been staple application areas for hollow core fibres.

### 4.1 Light Delivery

The delivery of UV/Vis light by hollow core fibres is one of the simplest and most effective uses of hollow core guidance. With the problems of loss and solarization in solid core fibres, hollow core fibres have already demonstrated their significant advantages in a number of reports [34, 45, 80]. The next generation of fibres will be able to build on these works and improve the loss, spectral range and modal content.

So far, the applications of ultraviolet guidance in hollow core has been over relatively short lengths < 100 m. If hollow core fibres can reach their predicted loss values [17] then delivery over 100–1000 m lengths becomes practical. This flexility in delivery length is not only useful for classical applications but also for single photon quantum sources, which frequently emit in the visible or ultraviolet. With successful demonstrations of quantum state delivery at near‐infrared wavelengths [81, 82] similar performance should also be possible at shorter wavelengths.

With reports of delivery at 343, 266 and 214 nm [34, 80, 83] hollow core fibres have shown delivery at ever shorter wavelength with energies not possible in solid core fibres. The extension to vacuum‐ultraviolet wavelengths, where even the air itself absorbs, has promising applications in space science [84, 85] and likely many others. The historical lack of vacuum‐ultraviolet guiding fibres makes predicting their impact difficult as entire fields have never considered that a flexible and low loss vacuum‐ultraviolet fibre might be possible. While the current work is in early stages, we have demonstrated transmission down to ∼140 nm [22] which includes the Thorium‐229 transition around 148 nm that has garnered significant recent interest [86].

Finally, in practically all reports of short‐wavelength delivery the fibres used were effectively single mode or used in that configuration. These fibre are perfect for coupling to an ideal single mode output but the reality is that many sources exhibit some degree of higher order mode content. To couple to multi‐mode or incoherent sources, a hollow core fibre with few or many modes is required. The same principles outlined in recent works [72, 87] should apply to shorter wavelengths and enable the efficient delivery of light from lower‐quality ultraviolet and visible laser beams.

### 4.2 Non‐Linear Generation

Optical fibres have long been excellent platforms for observing and controlling non‐linear interactions, owing to the tight confinement over long lengths that a fibre inherently provides. A common specific application is in the conversion of light from one wavelength to another, or to a range of wavelengths. In solid core fibres, these interactions are heavily constrained at UV/Vis wavelengths by challenging phase‐matching, high material losses and solarization. Despite the demonstrated potential of ZBLAN [88] glass, these issues have not been fully resolved in alternative glasses, and short‐wavelength generation remains difficult and limited in solid core optical fibres.

These limitations are bypassed in simple capillary fibres, which enable generation at intensities [89] and wavelengths [90] simply not possible in solid materials. However, with their guidance depending only on weak grazing incidence reflection [91], these capillary fibres require very large core diameters and must be kept completely straight for reasonable losses. To see any non‐linear interactions over these short straight lengths, they must be driven by high energy pulses from highly amplified laser systems, which limit their widespread adoption and use.

Anti‐resonant hollow core fibres represent an important bridge between solid core and simple capillary fibres, achieving capillary‐like guidance while remaining flexible and low loss at much smaller core diameters. This relaxes the pump energy constraints such that even a single gain managed non‐linear amplifier [92] is sufficient to drive non‐linear processes [93]. With a range of different non‐linear processes already demonstrated at near‐infrared and visible wavelengths [50, 93, 94], a future challenge will be extending these to ultraviolet wavelengths. At deep‐ultraviolet and vacuum‐ultraviolet wavelengths the previously discussed material limitations also affect non‐linear conversion in crystals and bulk material, meaning compact and bright sources are sorely needed.

For non‐linear generation at these short wavelengths, we require < 100 nm wall thicknesses to provide resonance free guidance in the fundamental band. These fibres can be driven by frequency converted pumps at 515, 343 nm which can be generated efficiently by second or third harmonic generation in bulk crystals. So far, a supercontinuum from 260–750 nm (at the 3 dB level) [73] has been demonstrated with ∼220 fs and 6 μJ input pulses at 515 nm. With a further reduction in wall thickness and pumping at 343 nm, a broadband VUV/UV supercontinuum source could even be possible. In a different scheme [93], tunable ultrashort pulses would also be possible across that same range at much lower energies than in current capillary systems [95].

If such reductions in wall thickness can be achieved, then much smaller core diameters should also be possible. These may enable the same non‐linear processes as large core diameters, but at much lower pulse energies, enabling higher repetition rates for the same pump power. The flexibility in core diameter is particularly large in nested structures [21], as the improved scaling of confinement loss allows for much smaller core diameters without sacrificing low loss guidance. With many reports of non‐linear generation using complex and expensive pump systems, reducing the pump laser power requirements should enable more practical and compact sources.

Many of these benefits have already been realized in post‐processed fibres [76, 77, 96], where challenging fibre parameters can more easily be achieved. However, the limited lengths of such fibres preclude many non‐linear interactions [49, 58, 97] that take place over several meters. By directly drawing fibres with comparable parameters, these interactions will be enabled, yielding a more diverse range of UV/Vis light sources. Furthermore, fibre fabrication is more scalable for manufacturing as it produces many meters of highly uniform fibre in one process, while tapering and etching require each short length to be individually produced.

## 5 Conclusion

Hollow core fibres represent a transformative opportunity for guiding light at UV/Vis wavelengths, offering performance far exceeding that possible in conventional solid‐core fibres. While their development has flourished at near‐ and mid‐infrared wavelength, progress toward shorter wavelengths has been constrained by fabrication challenges. This perspective has outlined the practical and fundamental fabrication bottlenecks that have hindered widespread adoption, while also identifying promising strategies that could unlock a new generation of low‐loss, broadband fibres.

If these fabrication advances are realized, hollow core fibres could enable long distance delivery of UV/Vis light, compact and efficient non‐linear light sources, and even practical guidance in the vacuum‐ultraviolet, a spectral region where no optical fibres currently exist. Their potential impact spans quantum optics, spectroscopy, biomedical imaging, and space science ‐ which have long awaited the maturation of hollow‐core fibre technology at shorter wavelengths.

## Conflicts of Interest

The authors declare no conflicts of interest.
